# A narrative exploration of psilocybin’s potential in mental health

**DOI:** 10.3389/fpsyt.2024.1429373

**Published:** 2024-10-30

**Authors:** Huitae Min, Soon Young Park, Jisu Park, Seongsu Na, Hoe-Suk Lee, Taejung Kim, Jungyeob Ham, Young-Tae Park

**Affiliations:** ^1^ Natural Product Research Center, Korea Institute of Science and Technology (KIST), Gangneung, Republic of Korea; ^2^ R&D Center, NeoCannBio Co., Ltd., Seoul, Republic of Korea; ^3^ Department of Biochemical Engineering, Gangneung-Wonju National University, Gangneung, Republic of Korea; ^4^ Division of Bio-Medical Science and Technology, University of Science and Technology (UST), Daejeon, Republic of Korea

**Keywords:** psilocybin, psychoactive, psychiatry, mental health disorders, dosing

## Abstract

Psilocybin, a psychoactive substance, has recently garnered attention for its high therapeutic potential in psychiatry. In this study, we investigated the multifaceted aspects of psilocybin, highlighting its chemical properties, mechanisms of action, and burgeoning role in psychiatric treatment. Furthermore, we examined the clinical applications and potential therapeutic benefits of psilocybin in the treatment of various mental health disorders, supported by accumulating clinical evidence. This review aims to deepen our understanding of the clinical impact of psilocybin, elucidate its therapeutic value, and propose directions for future research, thereby paving the way for its integration into mainstream psychiatric treatments. Psilocybin has been shown to be safe in clinical trials with manageable side effects. However, additional safety measures are required after this discussion, including dosing protocols, patient monitoring, and distress management strategies.

## 1 Introduction

Derived from various mushroom species, psilocybin has a complex history and exerts profound effects on human consciousness positioning itself as a compound with promising implications for modern medicine (1, 2). Its unique properties have attracted attention from various fields, including neuroscience, pharmacology, psychology, and anthropology. This review aims to highlight the value of psilocybin by exploring its discovery, mechanism of action, chemical synthesis, biosynthesis, industrial production, noteworthy uses, and cumulative clinical data.

The story of psilocybin commences with ancient traditions of indigenous cultures, where it was venerated as a sacrament and employed in rituals for altered states of consciousness, spiritual insight, and healing (3). This tradition brought psilocybin to the attention of the Western world through the research and investigations of pioneers and scholars, eventually leading to its laboratory synthesis and subsequent exploration.

Psilocybin has received considerable attention due to its potential in treating various mental health conditions, including depression, anxiety, and post-traumatic stress disorder (PTSD) (2, 4, 5). In recent years, psilocybin has secured its position in medicine and industry; therefore, it is important to find ways to make the synthesis of psilocybin more stable and efficient. In this review, we introduce the history and research on the chemistry and biosynthesis of psilocybin, its mechanism of action, and its clinical impact, and suggest future directions.

## 2 Mechanism of psilocybin action

It has been reported that psilocybin undergoes a series of metabolic processes in the body, ultimately transforming into psilocin, an active form with psychoactive effects (6). Psilocybin is absorbed by the stomach and intestines, entering the bloodstream. This process usually begins to take effect within 20–60 min of ingestion (7). The effects of psilocybin usually last for several hours, peaking approximately 2–3 h after ingestion. Alkaline phosphatase in the bloodstream converts psilocybin into psilocin. Psilocin is structurally similar to serotonin, a neurotransmitter involved in mood regulation that crosses the blood-brain barrier. Psilocin primarily affects the brain by binding to serotonin receptors, particularly 5-HT2A receptors (8, 9). This binding results in changes in serotonin transmission and signaling in various brain areas, particularly those related to mood and cognition. When psilocin binds to serotonin receptors, it alters normal patterns of neural activity in the brain, leading to characteristic hallucinogenic effects. These effects include changes in perception, mood, and thought patterns, as well as an increased sense of interconnectedness with the environment and others. Psilocin can also impact connectivity in various brain regions, potentially disrupting the default mode network (DMN) (10, 11). The DMN is a network of brain regions associated with self-directed thinking and sense of self. DMN disruption can lead to ego dissolution and an altered state of consciousness.

## 3 Biosynthesis of psilocybin

**Figure 1:**
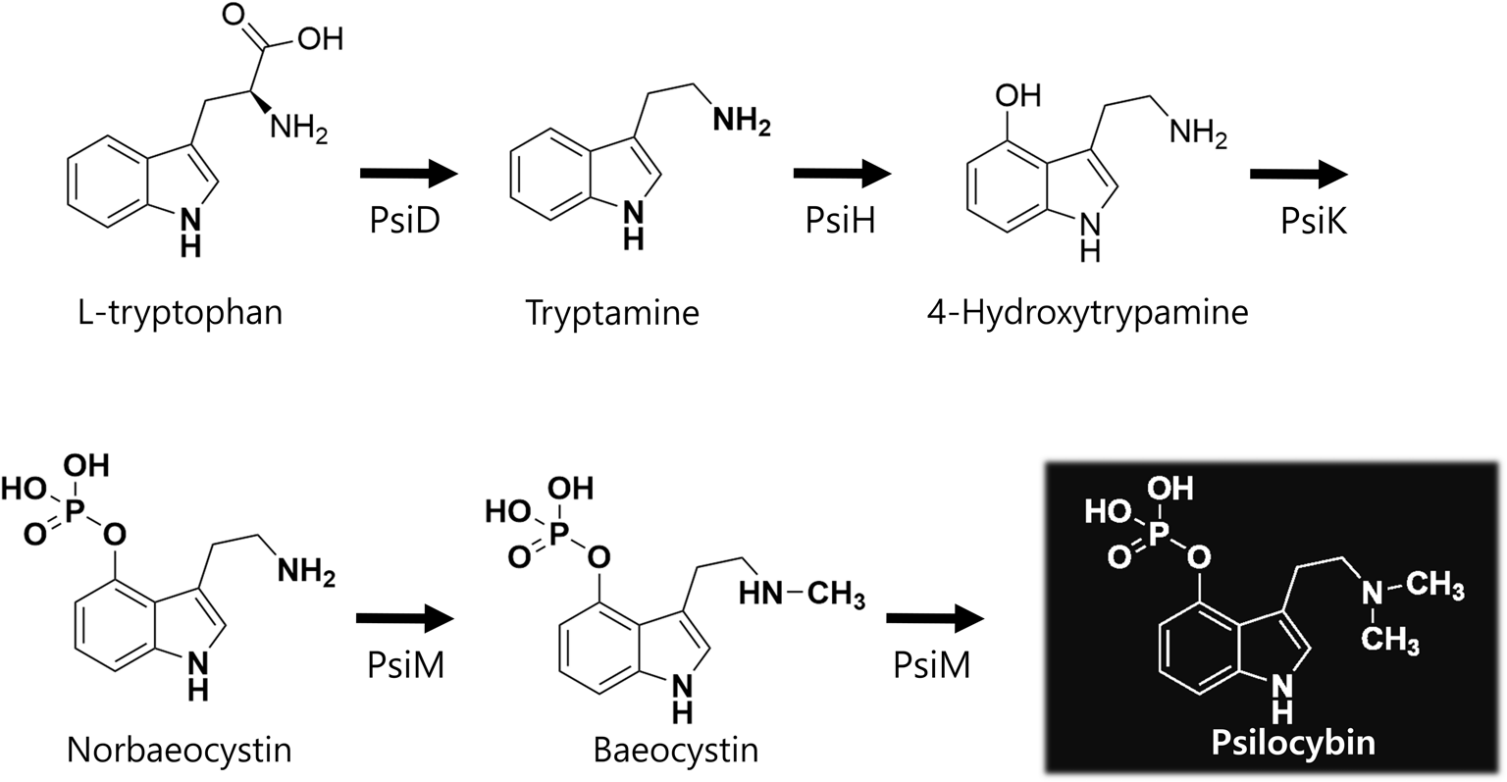
The pathway of biosynthesis of psilocybin.

Although research on the biosynthesis of psilocybin was limited before 2010, significant progress has been made in elucidating the biosynthetic pathways responsible for psilocybin production in psychedelic mushrooms, particularly *Psilocybe* species (12). This pathway involves enzymatic reactions that convert precursor compounds into psilocybins (**Figure 1**). Key enzymes crucial for psilocybin biosynthesis have been identified, with tryptophan decarboxylase (PsiD) playing a central role in the converting tryptophan to psilocybin. In 2017, four enzymes, PsiD, PsiH (4-hydroxylase), PsiK (kinase), and PsiM (methyltransferase), were introduced for the first time. Understanding the functions and mechanisms of these enzymes is important for elucidating their biosynthetic processes.

PsiD catalyzes the initial step in the psilocybin biosynthesis pathway by converting tryptophan to tryptamine (12–14). This reaction is critical because it removes the carboxyl group from tryptophan, setting the stage for subsequent modification. Following tryptamine formation, PsiH introduces a hydroxyl group at the 4-position of the tryptamine molecule, converting it into 4-hydroxytryptamine (also known as psilocin). This modification is vital for the hallucinogenic properties of psilocybin because hydroxylation significantly alters the interaction of the compound with neural receptors. PsiK phosphorylates 4-hydroxytryptamine by converting it into psilocybin. It attaches a phosphate group to hydroxylated tryptamine, creating psilocybin. This phosphorylation is essential for stabilizing the molecule and reducing its susceptibility to degradation before ingestion and conversion back into psilocin in the human body. In some pathways, PsiM is involved in the methylation of intermediates, contributing to the structural diversity of psilocybin. While the direct role of PsiM in the classic psilocybin pathway is less emphasized, methyltransferases are known to modify hallucinogenic compounds, potentially affecting their potency and psychoactive effects (14).

## 4 Therapeutic applications

The therapeutic applications of psilocybin, explored in a diverse range of clinical studies, have demonstrated its multifaceted potential in mental health treatment. We conducted comprehensive searches using databases such as PubMed and “ClinicalTrials.gov” in the National Center for Biotechnology Information (NCBI). The inclusion criteria included studies published between 2014 and the present, focusing on randomized controlled trials, open-label studies, and other clinical trials involving human participants. We excluded studies with significant methodological limitations, such as small sample sizes or the absence of appropriate control groups. To further assess the quality of the included studies, we employed the Cochrane Risk of Bias 2 (RoB 2) tool (**Supplementary Table 1**). The clinical studies, encompassing various conditions, dosages, and participant demographics, provide insight into how psilocybin can be used to address various mental health issues (**Table 1**). We describe the reports of psilocybin’s therapeutic effects across the following mental health issues.

**Table 1:** The table summarizing various studies on the effects of psilocybin for mental health issues.

Condition	Year	Participants	Dosing Information	Study Highlights
Mood	2014	25	Single dose of 0.16 mg/kg	Reduced amygdala reactivity to negative stimuli, correlating with increased positive mood
PTSD	2022	22	Single dose of 25 mg	No serious treatment-emergent adverse effects. Preliminary efficacy in facilitating confrontation with traumatic memories
	2022	20	Low dose (10 mg) on day 7, high dose (25 mg) on day 14 and optional dose (10 mg) on day 210	Ongoing phase 2 study, evaluating efficacy and safety
Combination with SSRIs	2023	19	Single dose of 25mg	A significant reduction in depression severity without serious adverse events
Social Decision- Making (with MDMA)	2018	19 for psilocybin, 20 for MDMA	Single dose of 2 mg (intravenous infusion)	Reduced rejection rates of unfair offers in economic game scenarios
Chronic Cluster Headaches	2024	10	Three doses (0.14 mg/kg)	Headache frequency was reduced by 30%
	2022	25	Low dose (0.0143 mg/kg or 1 mg), high dose (0.143 mg/kg or 10 mg), and placebo	A slight decrease in attack frequency during the first three weeks
Anorexia Nervosa	2021	10 Female	Single dose of 25 mg	Positive changes, including reduced anxiety and improved mood

### 4.1 Mood

Mood and depression are serious public health problems that cause a range of psychological and physiological complications. However, traditional treatments, such as selective serotonin reuptake inhibitors (SSRIs) and cognitive-behavioral therapy, although beneficial for many, do not provide adequate relief for a substantial subset of patients (15, 16). Recent studies have brought psilocybin to the forefront of potential alternative treatments for mood and depression (4). Its unique mechanism of action, primarily involving 5-HT2A receptors, differs significantly from conventional antidepressants, suggesting a potential for distinct and possibly superior therapeutic effects.

We used PubMed to search “Clinical Trial” articles from 2014 onwards containing both “psilocybin” and “mood” in the title. As the sole article on these results, the pivotal study involving 25 healthy volunteers explored the effect of psilocybin on amygdala reactivity (17). This study is crucial because the amygdala is known to play a significant role in emotional processing. This study found that psilocybin reduced the reactivity of the amygdala to negative stimuli, correlating with an increase in positive mood states among participants. This reduction in amygdala reactivity under the influence of psilocybin suggests its potential therapeutic application in the treatment of mood and depression, where emotional processing is often impaired. Complementing the findings on amygdala reactivity, many clinical trials focused on a significant challenge in mental health treatment: depression that does not respond to conventional antidepressants (18).

### 4.2 Treatment of PTSD

The potential of psilocybin in the treatment of PTSD is also a subject of growing interest. Its interaction with the serotonin 2A receptor is thought to result in altered states of consciousness and changes in emotional and cognitive processes. In PTSD, characterized by intrusive memories, hyperarousal, and emotional distress, psilocybin facilitates emotional processing and offers new perspectives on traumatic events.

Since there is no published “Clinical Trial” article on psilocybin for PTSD treatment, we searched “ClinicalTrials.gov” in the NCBI for relevant clinical data. There are two phase 2 clinical trials that are currently ongoing but not recruiting (ClinicalTrials.gov Identifier: NCT05312151, NCT05243329). The first study (NCT05312151) focused on evaluating synthetic psilocybin (COMP360) in 22 adults with trauma-induced PTSD. This open-label trial administered a single 25 mg dose of COMP360 and monitored participants over 12 weeks. Preliminary findings indicated that the treatment was well-tolerated, with no serious adverse effects reported, and showed potential efficacy in alleviating PTSD symptoms. Another study (NCT05243329) aimed to assess the efficacy of varying doses of psilocybin in individuals with PTSD. This randomized, placebo-controlled trial is still active, with an expected primary completion date in 2025. The study will provide valuable data on the long-term efficacy and safety of psilocybin for PTSD treatment.

In addition to this, a review article highlighted the need for innovative approaches in treating PTSD, citing the underwhelming efficacy rates of current treatments (19). It described an open-label study of traumatized AIDS survivors in which psilocybin-assisted psychotherapy (PAP) reduced PTSD symptoms, attachment anxiety, and demoralization. Several PAP trials have shown preliminary efficacy in facilitating confrontation with traumatic memories; decreasing emotional avoidance, depression, anxiety, pessimism, and disconnection from others; and increasing acceptance, self-compassion, and forgiveness of abusers–all relevant factors in PTSD recovery.

### 4.3 Psilocybin with SSRIs for depression

SSRIs are commonly prescribed antidepressants, however, many patients experience inadequate relief from these medications alone (16, 20). Recent studies have begun to explore the potential of combining psilocybin therapy with ongoing SSRI treatment to enhance therapeutic outcomes (21, 22).

We used PubMed to search “Clinical Trial” articles containing both “psilocybin” and “SSRI” in the title. There was only one report on the outcomes of COMP360 administered together with ongoing SSRI treatment in patients with depression (21). This research was particularly significant as it challenged the common practice of discontinuing antidepressants before administering psilocybin. The study involved 19 participants and found that COMP360 psilocybin therapy was generally well-tolerated when administered alongside SSRIs, showing a significant reduction in depression severity without serious adverse events. It suggested that the therapeutic potential of psilocybin was not hindered by concurrent SSRI treatment. These findings indicate that psilocybin therapy could potentially serve as an adjunctive treatment to SSRI antidepressants, challenging the previously held belief that SSRIs may interfere with the therapeutic effects of psilocybin. Furthermore, this finding is crucial in the context of treatment-resistant depression, in which patients may not respond adequately to traditional antidepressant treatments alone​​.

### 4.4 Psilocybin and 3,4-methylenedioxymethamphetamine in social decision-making

Social decision-making is notably affected in various mental health disorders due to altered cognitive processing and emotional regulation (23, 24). For instance, individuals with autism spectrum disorder might struggle with understanding social cues, and those with schizophrenia may face challenges in interpreting social interactions due to distorted perceptions. Similarly, anxiety and mood disorders like social anxiety disorder and major depressive disorder can lead to avoidance of social interactions, which further impairs decision-making in social contexts.

Several studies on psilocybin and 3,4-methylenedioxymethamphetamine (MDMA) in the context of social decision-making have provided intriguing insights into the effects of these substances on human behavior and cognitive processes (25, 26). We searched for “Clinical Trial” articles containing both “psilocybin” and “MDMA” in the title using PubMed. We reviewed the only published study that tested psilocybin and MDMA together in a clinical trial, which involved the Ultimatum Game, a standard economic game used to assess social decision-making and fairness (27). This game typically involves two players, one of whom divides the sum of money while the other chooses to accept or reject the offer. They found that both psilocybin and MDMA reduced the rejection rates of unfair offers. Specifically, when participants were under the influence of psilocybin compared with a drug-free session, there was a reduced probability of rejecting first-person unfair offers, with an odds ratio (OR) of 0.48, p=0.018. Similarly, for randomly generated unfair offers, the odds of rejection were also reduced, with an OR of 0.30, p=0.009, indicating a significant shift in decision-making favoring the acceptance of unfair offers. These findings are significant, offering insights into how psilocybin and MDMA may alter social cognition. By affecting the decision-making process in social contexts, especially in situations involving fairness and rewards, these substances can potentially be used to explore and treat disorders that affect social cognition.

### 4.5 Chronic cluster headaches

Cluster headaches are a severe type of headache disorder characterized by recurrent, unilateral pain, often around the eye, occurring in clusters lasting weeks or months with remission periods (28). Chronic cluster headaches (CCH) represent a more persistent and debilitating form, where headache episodes occur for more than one year without remission or with remission periods of less than one month (29).

Several studies and clinical trials have investigated the use of psilocybin for headaches and have shown promising results in terms of reducing the frequency and severity of attacks (30, 31). We searched for “Clinical Trial” articles containing both “psilocybin” and “CCH” in the title using PubMed. We reviewed the only published study examining clinical data on psilocybin in the treatment of CCH (32). It investigated the effects of three peroral doses (0.14 mg/kg) of psilocybin in CCH patients. The treatment was well tolerated without serious adverse reactions, and the attack frequency was reduced by 30% on average from baseline to follow-up. The study also reported a case of a patient experiencing complete remission for 21 weeks, highlighting the potential of psilocybin as a prophylactic treatment for CCH. The changes in hypothalamic functional connectivity observed in this study suggest neural pathway involvement in treatment response, indicating the need for further clinical studies to confirm the safety and prophylactic efficacy of psilocybin for CCH.

We searched “ClinicalTrials.gov” in the NCBI for relevant clinical data and identified two studies investigating psilocybin for cluster headache treatment. However, of these studies, only one has been completed. This completed study, sponsored by Yale University and collaborators, including the Heffter Research Institute and Ceruvia Lifesciences, examined the effects of an oral psilocybin pulse regimen (ClinicalTrials.gov Identifier: NCT02981173). The study design was interventional with a randomized crossover assignment and included 25 participants. The trial evaluated the safety and efficacy of psilocybin in treating headache disorders. Subjects were randomized to receive oral placebo, low-dose psilocybin (0.0143 mg/kg or 1 mg), or high-dose psilocybin (0.143 mg/kg or 10 mg) in three experimental sessions, each separated by 5 days. The primary outcomes included the time to the first and last attacks after the completion of the pulse regimen, changes in frequency, intensity, and duration of attacks, and health-related quality of life, among other measures. A randomized controlled trial using patient-informed, low-dose psilocybin pulse therapy demonstrated its safety with no unexpected serious side effects. A slight decrease in attack frequency was observed during the first three weeks, especially in patients with CCH.

Collectively, these studies provide valuable insights into the potential of psilocybin as a treatment for cluster headaches, especially in patients who have not experienced relief from existing therapies. These findings suggest that psilocybin could offer a novel approach to managing this challenging condition. However, further research is needed to fully understand its safety, efficacy, and long-term effects.

### 4.6 Anorexia nervosa

Anorexia nervosa (AN) is a severe and potentially life-threatening eating disorder characterized by an intense fear of weight gain, distorted body image, and severe restrictions on food intake (33, 34). Recently, psilocybin therapy has gained interest as an innovative treatment for AN (35). We searched for “Clinical Trial” articles containing both “psilocybin” and “anorexia nervosa” in the title using PubMed. From the results, we reviewed the only relevant published clinical study. This study recruited 10 participants who met the Diagnostic and Statistical Manual of Mental Disorders, Fifth Edition criteria for AN or pAN (partial remission) (36). They received a single 25 mg dose of COMP 360 in conjunction with psychological support. The adverse events were mild and transient, indicating that psilocybin therapy is safe and tolerable for female patients with AN. Furthermore, the average changes on Eating Disorder Examination subscales indicated that weight concerns decreased significantly from baseline (day-1) to 1-month (*P* = 0.036, Cohenʼs *d* = 0.78) and 3-month (*P* = 0.04, *d* = 0.78) follow-up. Significant decreases were observed in shape concerns at 1-month follow-up (*P* = 0.036, *d* = 0.78). Ninety percent of participants reported feeling more positive about life endeavors, and 80% considered the psilocybin session one of the most meaningful experiences of their lives. This trial indicated the possibility that psilocybin, combined with psychological support, was well-tolerated by participants, with some showing improvements in AN symptoms of anorexia nervosa, such as reduced anxiety and improved mood.

## 5 Impact on creative cognition

Research on the impact of psilocybin on creative cognition has produced intriguing findings suggesting that psilocybin can influence creative thinking processes (37). Studies of the neural effects of psilocybin have shown increased connectivity across various brain regions but not typically in direct communication (11, 38–41). This hyper-connectivity can lead to a state in which traditional cognitive boundaries are blurred, potentially facilitating expansive associative thinking. Such changes are hypothesized to underlie the reported increases in creativity and novel problem-solving abilities observed in some studies (37, 42, 43). These studies and anecdotal reports suggest that psilocybin can temporarily increase creative thinking during the psychedelic experience, and potentially for some time afterward. Several participants in these studies reported feeling more open to new ideas and were able to make unique connections between seemingly unrelated concepts. However, this research is still in its early stages, and more systematic investigations are needed to quantify these effects and fully understand their mechanisms of action.

## 6 Long-term emotional and brain function changes

Participants in psilocybin studies often report increased emotional openness, greater life satisfaction, and improved mood lasting for several weeks or even months following a single dose (44). These long-term benefits are thought to arise from intense, often introspective, experiences facilitated by psilocybin, leading to a re-evaluation of personal values, behaviors, and the resolution of past traumas. Moreover, psilocybin increased the connectivity between different brain regions, leading to enhanced communication across areas that do not typically interact (39, 40). This increased global brain connectivity is associated with novel thought patterns and cognitive flexibility reported by users after their experiences. These changes in brain connectivity have been hypothesized to underlie the lasting improvements in mood and cognitive function observed following psilocybin administration.

One study found that a single high dose of psilocybin significantly reduced negative affect and increased positive affect among participants (45). Measures of stress, anxiety, and mood disturbance showed considerable improvement one week after psilocybin administration, with some effects persisting for up to a month. Additionally, participants reported increased feelings of joy, content, pride, compassion, and amusement both one week and one-month post-treatment. These emotional shifts were accompanied by changes in brain function, particularly in the amygdala response to affective stimuli, indicating a reduction in the blood-oxygenation level-dependent response to all facial stimuli at one week compared to baseline, which then returned to baseline levels at one month.

Collectively, these studies suggest that psilocybin can facilitate profound and lasting changes in emotional openness, life satisfaction, and brain connectivity, potentially reshaping individuals’ outlook on life and enhancing their psychological resilience.

## 7 Safety and adverse events

Although psilocybin has demonstrated significant therapeutic potential in the treatment of various psychiatric disorders, it is important to consider its associated safety concerns and adverse events (7, 46). Physiologically, psilocybin can lead to temporary increases in heart rate and blood pressure, which may pose risks for individuals with pre-existing cardiovascular conditions. Therefore, caution is advised when considering psilocybin for patients with known cardiovascular issues. Moreover, psychological adverse events can include transient anxiety, confusion, or distress during the psilocybin experience (46, 47). In some cases, individuals may experience feelings of paranoia or have distressing hallucinations, commonly referred to as “bad trips”. Despite these potential adverse events, the available clinical data suggest that, when administered in a controlled therapeutic setting, the risk profile of psilocybin is generally manageable. However, further research is required to better understand the long-term safety of psilocybin, particularly in populations with underlying health conditions.

## 8 Discussion

Synthesizing the key findings from the studies on psilocybin, it is evident that psilocybin therapy holds significant promise for treating a range of psychiatric disorders, including depression, PTSD, anorexia nervosa, and cluster headaches. We highlight the capacity of psilocybin to induce profound changes in brain connectivity and cognitive flexibility, facilitating the re-evaluation of personal values and behaviors, often leading to improved mental health outcomes. These effects are attributed to the interaction of psilocybin with the serotonin system, which not only alters perception and mood but also enhances neural connectivity, enabling more flexible thought patterns and emotional responses (8–11).

We compared psilocybin therapy with conventional psychiatric treatments, highlighting its potential as a groundbreaking approach to mental health care. We underscored the efficacy of psilocybin, particularly for individuals unresponsive to standard treatments such as SSRIs, showing its ability to induce significant and lasting improvements in various conditions, including depression, PTSD, and anorexia nervosa. One standout point is the enduring nature of the benefits of psilocybin, such as enhanced emotional openness and cognitive flexibility, which persist beyond the immediate treatment period. This contrasts with the transient effects typically observed with conventional therapies. Moreover, the versatility of psilocybin in the treatment of a range of mental health disorders has been highlighted as a significant advantage over more narrowly focused treatments. Despite its potential, this study acknowledges the challenges of integrating psilocybin therapy into mainstream psychiatric practice, including the necessity for controlled administration environments, comprehensive psychological support, and further research to refine treatment protocols.

As evidence supports the efficacy and safety of psilocybin for treating various psychiatric disorders, there is an increasing call for a paradigm shift in mental health treatment approaches. This shift suggests a move towards more holistic and integrative therapies that address the root causes of mental health issues, rather than merely managing symptoms. Such changes would not only facilitate easier access to psilocybin for therapeutic purposes but also drive forward research into its potential benefits and applications. Moreover, the integration of psilocybin therapy into the existing healthcare systems is a significant future direction. Assessing the long-term outcomes of psilocybin therapy on individuals’ mental health and its broader societal impact is a key area of focus.
