# Supplementary material for: A narrative exploration of psilocybin’s potential in mental health
Source: Front Psychiatry. 2024 Oct 30;15:1429373. doi: 10.3389/fpsyt.2024.1429373 (PMC11557947; doi:10.3389/fpsyt.2024.1429373)
Supplement: Supplementary file 1 [file Table1.docx]

**Supplemental Material**

**A Narrative Exploration of Psilocybin’s Potential in Mental Health**

**Huitae Min^1^†, Soonyoung Park^2^†, Jisu Park^1,3^, Seongsu Na^1,3^, Hoe-Suk Lee^3^, Taejung Kim^1,4^, Jungyeob Ham^1,2,4^*, Young-Tae Park^1,4^***

**Affiliations:**

^1^Natural Product Research Center, Korea Institute of Science and Technology (KIST), Gangneung 25451, Republic of Korea

^2^NeoCannBio Co., Ltd., Seoul 02455, Republic of Korea

^3^Department of Biochemical Engineering, Gangneung-Wonju National University, Gangneung, 25457, Republic of Korea

^4^Division of Bio-Medical Science and Technology, University of Science and Technology (UST), Daejeon 34113, Republic of Korea

| **Condition** | **Reference** | **D1** | **D2** | **D3** | **D4** | **D5** | **Overall** |
| --- | --- | --- | --- | --- | --- | --- | --- |
| Mood | Kraehenmann et al., 2015 | 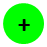 | 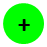 | 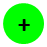 | 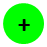 | 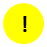 | 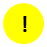 |
| PTSD | NCT05312151 | 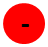 | 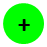 | 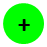 | 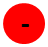 | 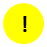 | 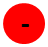 |
|  | NCT05243329 | 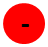 | 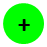 | 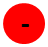 | 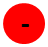 | 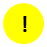 | 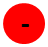 |
| Combination with SSRIs | Goodwin et al., 2023 | 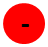 | 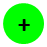 | 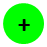 | 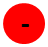 | 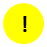 | 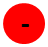 |
| Social Decision-Making (with MDMA) | Gabay et al., 2018 | 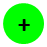 | 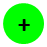 | 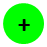 | 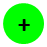 | 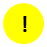 | 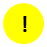 |
| Chronic Cluster Headaches | **Madsen et al., 2024** | 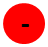 | 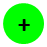 | 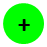 | 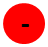 | 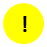 | 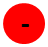 |
|  | NCT02981173 | 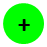 | 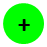 | 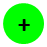 | 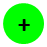 | 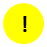 | 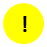 |
| Anorexia Nervosa | Peck et al., 2023 | 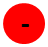 | 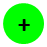 | 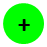 | 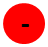 | 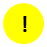 | 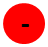 |

**Table S1. Risk of Bias (RoB) 2 assessment for the included clinical studies**

This table presents a detailed evaluation of the risk of bias in each clinical study included in this review, using the Cochrane Risk of Bias 2 (RoB 2) tool. The RoB 2 tool assessesed bias across five domains: (D1) randomisation process, (D2) deviations from the intended interventions, (D3) missing outcome data, (D4) measurement of the outcome, and (D5) selection of the reported result. Each domain is rated as low risk (+), high risk (-), or with some concerns (!).
